# Mortality among drowning rescuers in China, 2013: a review of 225 rescue incidents from the press

**DOI:** 10.1186/s12889-015-2010-0

**Published:** 2015-07-10

**Authors:** Yinchao Zhu, Xia Jiang, Hui Li, Fudong Li, Jieping Chen

**Affiliations:** Institute of Non-Communicable Disease Control and Prevention, Ningbo Municipal Center for Disease Control and Prevention, No. 237, Yongfeng Road, Haishu District, Ningbo, Zhejiang Province 315010 People’s Republic of China; Cardiovascular Epidemiology Unit, Institute of Environmental Medicine, Karolinska Institutet, Solna, 17172 Stockholm Sweden; Department of Public Health Surveillance & Advisory Division, Zhejiang Province Center for Disease Control and Prevention, No.630, Xincheng Road, Binjiang District, Hangzhou, Zhejiang Province 310051 People’s Republic of China

**Keywords:** Drowning, Rescue, Risk factors, Primary drowning victims, Victim-rescuer drowning incidents, Safety, China

## Abstract

**Background:**

Drowning is common worldwide. Rescue efforts attempted by untrained bystanders often lead to the death of the primary drowning victim (PDV), the rescuer or both. Our study aimed to inform prevention by identifying risk factors in rescuer drowning.

**Methods:**

Data on drowning rescue incidents reported online in mainland China, 2013, were reviewed. Information on the drowning incidents, PDVs and rescuers were retrieved for analysis.

**Results:**

A total of 225 rescue incidents were identified, of which 14 were victim-rescuer drowning incidents (VRDIs) (6.2 %). A person-to-person rescue by swimming to PDVs was the most commonly used method (58.9 %). Resuscitation was given immediately to 35.5 % of PDVs after rescue. The mortality rate of the rescuers (13.3 %) was similar to that of the PDVs (11.5 %) (*χ*^2^ = 0.5, *p* =0.49). Being an adult (OR = 0.2, 95 % CI: 0.1–0.5) and other than the first rescuer (OR = 0.4, 95 % CI: 0.2–0.9) decreased the risk of rescuers drowning.

**Conclusions:**

Most of the currently employed life-saving methods are dangerous and even potentially life threatening. The idea of “rescuers’ safety first” should be embraced, especially with teenage and child rescuers, who should never be encouraged to rescue others without first guaranteeing their own safety. Promotion of basic rescue skills should be implemented in the general public.

## Background

Drowning has been defined as the process of experiencing respiratory impairment from submersion or immersion in liquid [1], with outcomes classified as death, morbidity, or no morbidity [2]. Some survivors are left with permanent neurologic impairment [3, 4] that inflicts a heavy burden on both their family and society [5]. Moreover, drowning is a major public health concern and a leading cause of unintentional injury deaths globally [6–8]. The worldwide estimate of 388,000 drowning deaths in 2004 [8] is believed to be under-approximated by 40–50 % [9]. Because of preventive measures, death rates from drowning among high-income countries have declined over recent decades [6, 10, 11]. However, rates remain high among low- and middle-income countries [7, 8, 12, 13], and China ranks highest in the number of drowning deaths worldwide [8].

Drowning cases where bystanders were on the scene and made an effort to rescue (usually because of a sense of duty, supererogation and altruism [14]) often made a crucial difference in the victims’ survival [15]. Among the few studies focused on rescue-related drowning incidents in which the rescuers have drowned [14–17], the proportion of PDVs who survived has varied by population (43.9 % in Turkey and 93 % in Australia) [16, 17]. We actually do not know how many rescuers survived their rescue attempts. We only know those who lost their lives, but not an overall total number (rescuers who died plus rescuers who survived, as the baseline denominator); therefore, we cannot calculate the risk of drowning while attempting a rescue [14, 16, 17]. Moreover, given all the permutations of internal and external elements the rescuers would encounter, as well as the shortage of time to plan for a safe approach, untrained rescuers may not only fail to rescue victims, but may subject themselves to so-called victim-instead-of-rescuer syndrome [16] and cause multiple drowning incidents [17].

Therefore, the goal of this research was to inform development of preventive strategies by identifying the risk factors for drowning rescuers. The goal was addressed by examining the cases of drowning rescues reported online in the mainland Chinese media in 2013.

## Methods

**Fig. 1:**
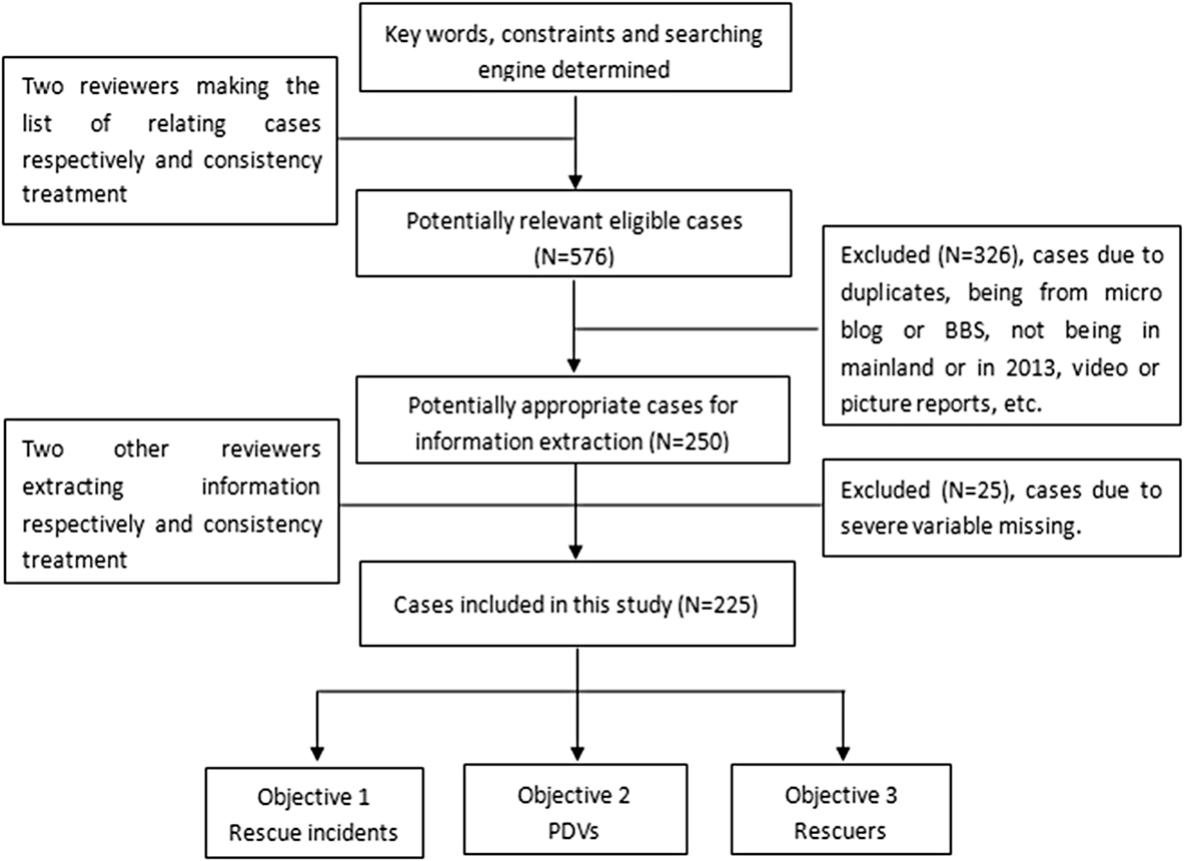
Flow chart of data collection process. The detailed peocess of data collection and quality control was showed

This study was approved by the ethical committee of Ningbo Municipal Center for Disease Control and Prevention. The data collection process is shown in a flowchart (Fig. 1). ‘Baidu’ (‘百度’ in Chinese), the world’s largest Chinese language search engine, was used to search for Chinese news reports. All cases were retrieved from online news reports with at least two of the following keywords tagged: ‘drowning’, ‘near-drowning’, ‘fall into water’, ‘rescue’, ‘life-saving’, and ‘sacrifice’ (‘淹死’, ‘溺水’, ‘落水’, ‘救人’, ‘救命’, ‘牺牲’ in Chinese). We covered both unintentional and intentional drowning cases. All the cases were from websites of national or regional newspapers, and police and fire departments. Owing to the lack of full information from a single report, related reports identified as having the same name of victims or rescuers or a similar news title were also searched further, with the aim of collecting the most complete information for each event.

The following variables were constructed: (1) Incidents: province, date, time, drowning locations; (2) PDVs: demographic characteristics, cause of drowning, whether reported by the news as being a swimmer, survival status, symptoms (for survivors) and whether first aid was provided; (3) Rescuers: demographic characteristics, whether reported by the news as being a swimmer, rescue rank (whether the rescuer was the first, second, or some subsequent person to attempt rescue), whether the rescuers called for others’ help and whether they undressed before the rescue, rescue measures, number of drowning victims in need, whether difficulties were experienced and the nature of such difficulties, and survival status. After excluding 25 cases that were missing more than half of the variables, we eventually obtained 91 cases of incidents, 99 PDVs, and 64 rescuers with virtually complete information, constituting 40.4 %, 36.5 % and 15.4 % of the total number of incidents (225), PDVs (271) and rescuers (416), respectively.

### Terms and definitions

The details on terms and their definitions are provided in Table 1. In addition, season was defined on the basis of Chinese meteorological phenomena (spring: Mar, Apr and May; summer: Jun, Jul and Aug; autumn: Sep, Oct and Nov; winter: Dec, Jan and Feb). Time was divided into four sections (Night: 0:00–6:00; Morning: 6:00–12:00; Afternoon: 12:00–18:00; Evening: 18:00–24:00). The target population was classified into two groups (children/teenagers: <18; adults: ≥18). All variables related to the rescue incidents and rescuers were defined by the authors of the present study.

**Table 1:** List of terms and definitions

Terms	Abbreviations	Definition
Primary drowning victim	PDV	PDV referred to the victim who fell into liquid initially and may result in death from drowning [17].
Rescue		Rescue meant rescuers managed to save the original PDV and any rescuers who became PDVs directly no matter they were dead or survival finally.
Victim-rescuer drowning incident	VRDI	VRDI meant rescuers became dead as well as PDV in the rescue incident.
Survival		Survival was defined as a PDV and/or rescuer who had vital signs or regaining them after resuscitation as judged by reviewers based on the media reports.
Successful rescue		Successful rescue meant rescuers helped the drowning victim to extricate from liquid.
Rescue rank		Rescue Rank is the order for each rescuer who undertakes a rescue action, a rank of ‘1’ would be given to the first person who carried out the rescue, ‘2’for the next, and so on. If several rescuers implemented simultaneously, the same rank would be given to each.

### Data analysis

All data were analyzed in SAS V9.20 (SAS Institute, Inc.; Cary, North Carolina, USA). The Pearson *χ*^2^ test and Kruskal–Wallis rank-sum test with two independent samples were used for categorical and continuous outcomes, respectively. The data for number of rescuers and rescuer deaths were clustered and showed negative binomial distributions. Therefore, a two-level unconditional logistic regression model was adopted for the multivariate analysis. A *p*-value of less than 0.05 was set as the level of significance in this study.

## Results

### Rescue incidents

In total, 225 rescue-related incidents were identified from January 1 to December 31, 2013, including 271 PDVs and 416 rescuers, from 32 provinces, autonomous regions or municipalities in mainland China. The three provinces with the greatest share of incidents were Zhejiang (33, 14.7 %), Jiangsu (25, 11.1 %) and Sichuan (20, 8.9 %). Incidents varied by seasons, with a larger proportion identified in spring, summer and autumn (21.8 %, 37.3 % and 30.2 %) than in winter (10.7 %). Almost all cases occurred during daytime (22.5 % in the morning and 55.0 % in the afternoon), with a small proportion occurring during the night (1.5 %) or evening (21.0 %).

Twenty eight (12.4 %) rescue incidents had more than one PDV, with a maximum of five. In just over half (53.0 %) of cases, PDVs were adults only; in 44.2 % they were children only and in 2.8 %, both adult and child PDVs were identified. In 54.2 % of cases there was only one rescuer, in 26.7 % there were two, in 9.3 % there were three, and in 9.8 % there were more than three, with a maximum of 12. The average number of rescuers in a rescue incident surpassed that of PDVs (1.9 versus 1.2, *p* < 0.01). There were 28 incidents (12.4 %) with at least one PDV mortality, and 40 incidents (17.8 %) with at least one rescuer mortality; together these contributed to 14 VRDIs (6.2 %). Furthermore, there were 26 cases (11.6 %) with no PDV mortality but more than one rescuer mortality, 14 cases (6.2 %) with no rescuer mortality but more than one PDV mortality, and 171 incidents (76.0 %) with no mortality.

### Primary drowning victims

The successful rescue rate for PDVs was 88.5 % (239 of a total of 270 PDVs) (Table 2); 55.4 % were males, 54.2 % were adults and 91.9 % were non-swimmers (Table 2). The majority (96.3 %) of the drowning incidents occurred in fresh water. Ponds were the most common drowning places for children but not adults (18.6 % versus 2.8 %, *χ*^2^ = 20.3, p <0.01). Falling was the first leading cause of drowning, accounting for 64.6 % of incidents (Table 3). After being rescued, 56.8 % of PDVs survived with no obvious symptoms; 27.2 % were in a coma; 9.6 % experienced apnea or dyspnea and 2.6 % went into cardiac arrest. Among all PDVs, 35.5 % were given pre-hospital resuscitation on the scene, and 39.9 % were sent to hospitals without delay (data not shown).

**Table 2:** Basic information on PDVs and rescuers in 225 rescue incidents, n (%)

	PDVs	Rescuers	Chi-Square	*p*-value
Total	271	416		
Average number of persons per incident	1.2	1.9	59.9	<0.01
Gender				
Male	139(55.4)	373(91.0)	113.0	<0.01
Female	112(44.6)	37(9.0)		
Total	251	410		
Being an adult				
Yes	141(54.2)	337(81.6)	58.1	<0.01
No	119(45.8)	76(18.4)		
Total	260	413		
Being a swimmer				
Yes	18(8.1)	251(79.9)	269.4	<0.01
No	205(91.9)	63(20.1)		
Total	223	314		
Being a survivor				
Yes	239(88.5)	359(86.7)	0.5	0.49
No	31(11.5)	55(13.3)		
Total	270	414		

The inconsistencies among the total frequencies of certain variables were caused by missing data

**Table 3:** Drowning cause and locations among 271 PDVs

	N	(%)
Locations of drowning		
River/creek/stream	176	65.7
Lake	31	11.6
Pond	26	9.7
Ditch	14	5.2
Other (*e.g.,* reservoir, well, swimming pool)	11	4.1
Sea/beach	10	3.7
Total	268	100.0
Cause of drowning		
Falling from the bank	142	64.6
Traffic accident	30	13.6
Suicide	18	8.2
Other (*e.g.,* swimming accident, flood, electric shock)	11	5.0
Row/raft	10	4.6
Sliding off in the water close to the shore	9	4.1
Total	220	100.0

The inconsistencies among the total frequencies of certain variables were caused by missing data

### Rescuers

The average number of drowning people in need of rescue (including the original PDV and any rescuers who became PDVs) was 1.5 ± 1.0 per incident. Despite a relatively high successful rescue rate among PDVs, no significant difference in mortality rate was found between rescuers (13.3 %) and original PDVs (11.5 %) (Table 2). Compared with PDVs, rescuers were more likely to be males (91.0 %), adults (81.6 %) and swimmers (79.9 %) (Table 2). Significantly fewer (38.6 % versus 86.9 %) child than adult rescuers were swimmers (*χ*^2^ = 55.4, p < 0.01).

For rescuers, 12.1 % asked for assistance from others and 26.8 % removed either their shoes or clothing before the rescue. Most of the rescuers (80.1 %) chose to enter the water in the attempt to save the victims. Furthermore, 58.9 % of rescuers swam to PDVs to undertake rescue directly. Owing to lack of swimming skills, exhaustion or difficult water conditions, 97 rescuers (23.5 %) encountered hazardous situations and experienced difficulties; of these, 57.3 % died (Table 4).

**Table 4:** Rescue methods and causes of experiencing difficulties among 416 rescuers

	N	(%)
Rescue methods		
Swimming to the victim	245	58.9
Pulling in water close to the shore	88	21.2
Giving an extension(*e.g.,* pole, lifeline)	45	10.8
Pulling on shore	34	8.2
Pulling by standing on the boat	19	4.6
Throw the floats	18	4.3
Total	416	
Cause of experiencing difficulties		
Being a non-swimmer	36	37.1
Exhaustion	30	30.9
Bad water conditions (*e.g.,* torrent, turbulence)	28	28.9
Steep edge	13	13.4
Other (*e.g.,* cold, electric shock)	7	7.2
Total	97	

The average rescue rank was 1.5 ± 0.8. The rescue rank for those who died was significantly lower than for those who survived (1.3 versus 1.5, *p* < 0.01). A significant association between location and likelihood of rescuer survival was identified, wherein rescuers in a lake were less likely to drown compared with those in ponds and other locations. Adult and swimmer rescuers were more likely to survive than children and non-swimmers. Rescuers who used only their hands to pull victims through the water to shore had the lowest survival rate, and those who performed rescues in the context of a boating situation (pulling the victim by standing on the boat) the highest (Table 5).

**Table 5:** Factors in rescuer survival and mortality, n (%)

	Survival	Dead	Chi-Square	*p*-value
Total	359	55		
Average rescue rank^a^	1.5	1.3	6.7	<0.01
Average number of drowning men in need of rescue^a^	1.5	1.3	10246.5	0.08
Season				
Spring	78(21.7)	13(23.6)	2.1	0.55
Summer	128(35.7)	24(43.6)		
Autumn	116(32.3)	13(23.6)		
Winter	37(10.3)	5(90.1)		
Time				
Night	4(1.2)	0(0.0)	1.5	0.69
Morning	73(22.3)	14(27.5)		
Afternoon	193(59.0)	30(58.8)		
Evening	57(17.4)	7(13.7)		
Location				
River/creek/stream	228(64.4)	30(54.6)	25.1	<0.01
Lake	47(13.3)	2(3.6)		
Pond	29(8.2)	12(21.8)		
Ditch	17(4.8)	1(1.8)		
Other (*e.g.,* reservoir, well, swimming pool)	17(4.8)	9(16.4)		
Sea/beach	16(4.5)	1(1.8)		
Gender				
Male	321(86.5)	50(13.5)	0.1	0.07
Female	36(97.3)	1(2.7)		
Being an adult				
Yes	307(91.4)	29(8.6)	35.9	<0.01
No	49(65.3)	26(34.7)		
Being a swimmer				
Yes	228(91.2)	22(8.8)	22.8	<0.01
No	43(68.3)	20(31.8)		
Calling for others’ help when rescuing				
Yes	45(91.8)	4(8.2)	0.9	0.35
No	303(85.8)	50(14.2)		
Undressing when rescuing				
Yes	82(89.1)	10(10.9)	0.1	0.92
No	221(88.8)	28(11.2)		
Rescue methods				
Pulling on shore				
Yes	32(94.1)	2(5.9)	0.1	0.29
No	327(86.1)	53(13.9)		
Pulling in water close to the shore				
Yes	64(73.6)	23(26.4)	16.5	<0.01
No	295(90.2)	32(9.8)		
Swimming				
Yes	215(88.1)	29(11.9)	1.0	0.31
No	144(84.7)	26(15.3)		
Throwing the floats				
Yes	17(94.4)	1(5.6)	0.2	0.49
No	342(86.4)	54(13.6)		
Giving an extension				
Yes	44(97.8)	1(2.2)	5.4	0.02
No	315(85.4)	54(14.6)		
Pulling by standing on the boat				
Yes	19(100.0)	0(0.0)	0.1	0.09
No	339(86.0)	55(14.0)		

^a^indicates two independent samples Kruskal–Wallis rank sum test was used for analysis. All other analyses were Pearson *χ* ^2^ tests

After adjustment for confounding factors in a two-level logistic regression, only two factors remained significant as predictors of rescuer drowning. Being an adult rescuer was associated with an 83.6 % decrease in the risk of drowning (AOR = 0.2, 95 % CI: 0.1–0.5, *p* < 0.01), while increasing rescue rank predicted a 55.7 % decrease in risk (AOR = 0.4, 95 % CI: 0.2–0.9, *p* < 0.05) (Table 6).

**Table 6:** Two-level logistic regression analysis for risk factors associated with rescuers’ drowning

	Regression coefficient β	*p*-value	Odds Ratio (95 % CI)
Rescue rank	−0.8	<0.05	0.4(0.2–0.9)
Being an adult	–1.8	<0.01	0.2(0.1–0.5)
Being a swimmer	1.3	0.06	3.6(0.9–14.4)
Pulling in water close to the shore	0.2	0.82	1.2(0.3–4.9)
Giving an extension	–1.7	0.24	0.2(0.1–3.1)
Location			
Lake	−0.9	0.37	0.4(0.1–2.8)
Pond	1.3	0.08	3.7(0.9–16.3)
Other (*e.g.*, reservoir, well, swimming pool)	1.5	0.07	4.7(0.9–25.7)

## Discussion

As a populous developing country, China not only ranks highest in the number of drowning deaths worldwide [8] but also has a high drowning mortality rate of 4.4 per 100,000 people [13]. Among drowning deaths in China, some would certainly be cases of victim-instead-of-rescuer syndrome, although the proportion is unclear as there is a lack of relevant research. Our data suggest an average of 0.2 (55/225) rescuer deaths per rescue incident, and a mortality rate of rescuers statistically indistinguishable from that of PDVs, which indicates that drowning rescue is a high-risk activity [18, 19]. Ensuring awareness of rescuers’ safety first [20] might help reduce the mortality.

Unfortunately, but perhaps unsurprisingly, children constituted a large proportion of drowning victims for both PDVs [6, 21] and rescuers. The underlying reasons could be diverse. On one hand, children usually have limited competence at flexible cognitive control, exhibit a low risk perception and an overestimation of their swimming ability, all of which could lead to increased immature decision-making and risk-taking behaviors [22–24]. This speculation may be supported by the fact that more non-swimmers were found among the children than among the adult rescuers in our current study. On the other hand, the ethics that children have learned could also drive them to take altruistic actions [14, 25]. Therefore, the appropriateness of firmly discouraging children who cannot swim from rescuing should be brought into public awareness.

Local geographic conditions accounted for most of the variability in drowning incidence [2]. The top three provinces involved in our study, which were not the provinces with the largest populations, were all located within the Yangtze River Basin, which is known for its large number of various bodies of water. Consistent with other reports in China, most of the rescue incidents in our study occurred in fresh water and natural bodies of water [26, 27]. Our results indicated that drowning location had a critical impact on the rescuers’ survival, wherein lakes appeared to present lower risk than ponds, reservoirs and wells. Possible explanations for this location difference could be multifactorial. The lakes, whether artificial or natural, attract sightseers; therefore, multiple witnesses are typically available to perform rescue in a collaborative way. In contrast, ponds, reservoirs and wells in China often have steep and slippery water edges with considerable depth, resulting in lengthier stays in the water, along with greater difficulties for rescuers, all of which lead to detrimental outcomes in general [28]. A study conducted by Moran [29] also revealed that it was difficult for persons to exit deep water with variable edges. We further found that age was associated with the drowning location [2]; *i.e.*, ponds were particularly high-risk places for children. However, children as PDVs also seem particularly likely to be associated with rescuer fatality [16], although the reason for this is unclear. Possible explanations could be that children are incapable of providing good cooperation to the rescuers, and that their rescuer was likely to be a peer.

Rescue efforts in hazardous conditions were often highly challenging, especially when additional rescuers or tools were not readily available [18, 28]. Pearn *et al.* indicated that 50 % of rescuers in rescuer-who-drowns incidents were not familiar with the particular body of water and its hazards [14]. Therefore, drowning location would be a key variable in drowning prevention for both PDVs [2] and rescuers. Good understanding of the water environment before rescue should be strongly emphasized, and plans to effectively implement rescue operations in likely drowning environments need to be in place. Additionally, placement of isolation fencing and simple life-saving instruments in open water areas presumed to pose a high drowning risk could not only prevent most of the drowning caused by falls [2] but also provide PDVs and rescuers with floatation objects to hold that would improve the chances of survival [30].

Although the causes of drowning among rescuers were complex, several preventive suggestions have been proposed [16, 17]. A successful rescue is technically challenging [18, 19]. Rescuers without prior rescue knowledge mostly blundered into dangers and encountered life-threatening events, including death [17]. Our study showed that pulling PDVs directly in the water increased the likelihood of a drowning fatality for rescuers. This may be owing to the fact that rescuers standing in water are more likely to lose their balance and become entangled with PDVs; this typically occurs with a contact tow (a rescuer human chain) [31]. Other practices such as swimming to the victim have also been found to be dangerous [14, 15]. Therefore, direct personal contact in an aquatic environment is extremely dangerous for rescuers. It has been proposed that rescue attempts from land or boat be the top priority [32]. The employing of water-away and indirect contact (offering an object such as a pole, stick, rope or plank)—or even non-contact—was the secondary recommendation [30, 33, 34]. In addition, some professional rescue tools, such as rescue buoys, lifejackets, boats and lifelines, have proven essential for successful aquatic lifesaving [18, 19]. Unfortunately, few Chinese rescuers performed water rescues properly.

On-scene rescuers also assume the responsibility to perform on-site pre-hospital resuscitation after rescue [35]. It has been reported that almost 30 % of PDVs who were rescued by bystanders needed cardiopulmonary resuscitation [15], which, if done properly, could effectively contribute to a positive outcome [36, 37]. Rates of on-scene resuscitation provided by bystanders vary among studies [15, 36, 38]. A Dutch study showed that approximately 31.8 % of victims received resuscitation aid from bystanders [15]. Similarly, Topjian *et al.* found that one-third to one-half of rescued children in their study had received bystander cardiopulmonary resuscitation [38]. A hospital-based prospective study in Japan reported that 92 % of the patients transported to hospital because of drowning had been given pre-hospital resuscitation [39]. The rate of pre-hospital resuscitation among victims in China is lower than these figures, leaving substantial room for improvement. Therefore, educating the public in basic first aid skills could be an important step [30].

### Limitations

Although the present study offers a valuable data source for exploring drowning rescue events because most of the key variable information was contained in the reports [17, 40–42], the media is likely to underestimate the number of cases because of selective reporting [41]. Therefore, it is impossible to provide population-based rates on either PDVs or rescuers, and without population data it is not possible to extrapolate this information to all drowning rescue attempts. The media articles also often lack information on potential confounds, such as characteristics of water flow, the distance from shore, alcohol consumption of the rescuers, life vest application, differences in estimated swimming capacity (*e.g.*, weak, average or strong), relationship between victims and rescuers, whether victims were warned of the potential drowning peril, or how the call for help was made. Moreover, we could not rule out the possibility that rescued victims died later, because media reports typically reported on victims’ deaths at the scene but lacked post-rescue follow-ups. Additionally, missing data was another unavoidable problem: in cases where the rescuers left immediately after the rescue action, or where many rescuers performed rescue simultaneously, it would be difficult for the media to collect information on each of the rescuers at the desired level of detail.

## Conclusions

Despite the survival of most PDVs and their rescuers, drowning rescue incidents still constitute a large number of unwanted VRDIs. A victim’s young age (*e.g.*, being a child) increased the risk of the rescuer’s death. In China, drowning locations influenced rescuers’ survival, and most of the life-saving methods employed were dangerous for rescuers. Moreover, the subsequent rate of on-scene resuscitation after a successful rescue was far from sufficient. Accordingly, the idea of rescuers’ safety first and discouraging children from rescue should be proposed. The results suggest the necessity of promoting education and training for the public to develop basic skills for a successful rescue, such as risk evaluation, request for bystanders’ support, preference for non-contact rescue strategies and skills of post-rescue resuscitation.
